# Digital collaborative learning: identifying what students value

**DOI:** 10.12688/f1000research.6223.1

**Published:** 2015-03-20

**Authors:** Claire Hemingway, Catrina Adams, Molly Stuhlsatz

**Affiliations:** 1 National Science Foundation, Arlington, Virginia, 22204, USA; 2 Botanical Society of America, St. Louis, Missouri, 63166, USA; 3 Biological Science Curriculum Study, Colorado Springs, Colorado, 80918, USA

**Keywords:** Attitudes, Plant Science, E-mentoring, Authentic Science, Student-Teacher-Scientist Partnership

## Abstract

Digital technologies are changing the learning landscape and connecting classrooms to learning environments beyond the school walls.  Online collaborations among students, teachers, and scientists are new opportunities for authentic science experiences.  Here we present findings generated on PlantingScience ( www.plantingscience.org), an online community where scientists from more than 14 scientific societies have mentored over 14,000 secondary school students as they design and think through their own team investigations on plant biology.  The core intervention is online discourse between student teams and scientist mentors to enhance classroom-based plant investigations.  We asked: (1) what attitudes about engaging in authentic science do students reveal, and (2) how do student attitudes relate to design principles of the program? Lexical analysis of open-ended survey questions revealed that students most highly value working with plants and scientists.  By examining student responses to this cognitive apprenticeship model, we provide new perspectives on the importance of the personal relationships students form with scientists and plants when working as members of a research community. These perspectives have implications for plant science instruction and e-mentoring programs.

## Introduction

A revolution in digital learning is underway. The number of students taking online courses in the United States has skyrocketed to 7.1 million in higher education institutions ( Allen & Seaman, 2014) and almost 750,000 in public primary and secondary schools ( Evergreen Research Group, 2014). Digital technologies offer new mechanisms to support reform-based approaches and increase student engagement. Transforming traditional college and pre-college classrooms into active-learning environments where students interact with peers and instructors to collectively construct and apply knowledge can positively impact student attitudes towards science ( Armbruster *et al.*, 2009; Gibson & Chase, 2002; Taraban *et al.*, 2007; Ward *et al.*, 2014). A significant challenge to the tremendous potential for the digital learning revolution is transferring authentic science investigations to digital learning environments. There is a particular need to investigate students’ attitudes about technology-enhanced science investigations in precollege settings, as this is a critical time when interest in science can set the direction of future career goals ( Maltese & Tai, 2011).

How students and teachers experience plant science is a focus of concern; alarming trends in U.S. formal education show that plants are under-represented in teaching materials, and poorly understood. A decline in botanical literacy is part of the continuing U.S. crisis in science literacy, although some underlying causes are unique to botany. The best-selling U.S. high school biology texts feature primarily animals ( Uno, 1994). Teachers also place a focus on animals; when choosing material to teach biological concepts, teachers reported preferring to use animal examples over plant examples ( Flannery, 1999; Link-Perez & Schussler, 2013). Pre-service teachers ( Krantz & Barrow, 2006) and young learners ( Barman *et al.*, 2006) hold many of the same misconceptions (or alternate conceptions) about plants. High school biology teachers from across the U.S. report being least confident about plant biology when surveyed about five fundamental topics, and just 46% of those with 6 years or less teaching experience report having ever had a botany course ( Horizon Research Inc., 2002; Horizon Research Inc., 2013). The problem is not restricted to the U.S. Research on the uptake of plant sciences in the United Kingdom shows that the majority of UK students entering university biology courses have little interest in or knowledge of plants ( Stagg *et al.*, 2009).

Compounding these documented issues is the human tendency to overlook plants, known as ‘plant blindness’ ( Wandersee & Schussler, 1999), which has both cultural and physiological underpinnings ( Balas & Momsen, 2014). Educators, students, and the public who generally don’t notice plants in the environment are not likely to see that plants are of utmost importance to the food, fuel, fibers and pharmacology of everyday life, as well as the functioning of our global ecosystem. A future workforce prepared with an understanding of plant science and cross-cutting concepts applied in innovative solutions will be needed to meet societal challenges, such as coping with climate change, feeding an increasing population, and generating sustainable energy sources ( National Research Council, 2009). Engaging scientists as mentors has the potential to inspire interest and to link classroom learning to real-world authentic science. For plant science, meaningful and early exposure may be critical:

*“The presence of a plant mentor earlier in one’s life (someone who helped the mentee observe, plant, grow, and tend living plants) is a key predictor of that person’s awareness, appreciation, and understanding of plants throughout the lifespan.”* ( Wandersee & Clary, 2006).

Given the need to enhance teaching and learning about plants in formal U.S. education and the promise of student-scientist partnerships ( Houseal *et al.*, 2014; Summers & Hrabowski, 2006), we have been engaged in an approach to foster student learning of scientific practices and plant biology through interactions with scientist mentors. The PlantingScience program was intentionally developed as a blended approach to student-centered experiential learning taking place in the classroom, supplemented by communication and collaboration with peers and experts online. The online platform ( www.plantingscience.org) not only eliminates geographic limitations, its design features make student thinking visible, enabling students, teachers and mentors to monitor thinking and learning and provide feedback. An impetus for this study was to take a systems approach to examining the inputs and outputs of PlantingScience. A previous study examined the techniques mentors used in online discourse with student teams ( Adams & Hemingway, 2014). Here we examine the attitudes (affective responses) of students participating in collaborative plant investigations. We present qualitative data on what students value about a digital learning environment in which science practices and content are integrated and science experts and novices collaborate, as it occurs in authentic science research. We ask what major themes emerge from the student responses and how the exploratory analyses relate to design features of the program.

## Methods

### Context of PlantingScience

Students ages 11 to 18 are the focus of this study. The students mentored by volunteer scientists enter the program through their teachers, who typically are seeking inquiry learning opportunities for their students. Participating classrooms (60% high school, 40% middle school) come from a variety of demographics; rural, urban, public and private schools. Classroom teachers choose one of the eight available investigation themes and decide whether the 3–12 week long projects would be limited to controlled experimentation or include observational studies; their past experience with inquiry often determines how guided or open student projects will be. The research questions and plants used by student teams vary widely; however, all investigations intend for students to collaboratively develop a research question on a core idea in plant biology, plan and carry out an investigation to answer the question, analyze the data, and make sense of the findings.

Each student team is assigned a unique project page where they are encouraged to post information about their research project, as well as engage in asynchronous dialog with the mentor matched to their team. The program’s 988 registered mentors, from undergraduate students to professor emeriti, belong to more than 14 scientific societies that partner in the program. Student pre- and post-tests are not mandatory, and they are administered through the online platform, which is a customization of the open-source content management system Zikula. The Institutional Review Board of Texas A&M University granted approval for collection of these data, and we obtained permission from schools, students, and parents where appropriate, for publication. Over 4000 team projects with associated dialog, archived since 2005 are available at www.plantingscience.org.

### Data sources

For this study we analyzed students’ open-ended responses to the post-test survey question, *“What did you like most about this experience?”* Following six online mentored inquiry sessions between 2010 and 2012, only 2.7% of the students who initiated the online survey did not complete the open-ended question. A total of 2,617 responses from middle school (n = 947) and high school (n = 1,670) students were analyzed. The students completing surveys over this period were in classrooms of 20 middle school and 42 high school teachers. These classrooms encompassed a range of private and public schools, including two international classrooms. As expected given that “Wonder of Seeds” is the most frequently used module, more than half (54.6%) of the students who completed the surveys had conducted germination and/or seedling growth studies.

Student Responses Anonymized, raw data of student responses to optional open-ended survey question administered online following secondary school students’ participation in the online mentored inquiry experience.

### Analysis of student attitudes

We downloaded the student responses from the archives in the online platform, removed errant duplications, and then imported an aggregated file into IBM ^®^ SPSS ^®^ Text Analytics for Surveys version 4 (IBM copyright 2010). As responses to the open-ended survey question typically ranged from one to several sentences, the computational linguistics text mining tool simplified the creation of broad sets of categories across responses. From the initial automated categorization of results, we discussed refinement to the categories through several iterations. In particular, we identified where automated codes were not applied appropriately, automated categories were conceptually related, and custom terms needed. For example, student comments about observations, collection of data, measurement, and analysis of data were manually grouped in an overarching category on the practice and processes of science. Similarly, text referring to seeds and germination were grouped, and the students’ various descriptions of doing experiments, labs, or projects were defined as synonyms. This iterative, exploratory process results in a robust view of the elements of most interest to the students as well as allowing one to investigate connections between areas of interest.

## Results

To put the student survey data in perspective, we first present statistics on content of the project pages as a way to quantify the student experience and to provide some context of what the experience involved during the period investigated here ( Table 1). Most student projects included information on the team’s research question, prediction, experimental design and conclusion. Projects variably included supplemental documentation about their team research. The number of asynchronous posts between student teams and mentors ranged widely. There are many factors that account for the wide range in student post numbers, from teachers’ directions whether all students should post or appoint a team spokesperson, to the number of days computers are available, to teachers’ grading structures, to individual student motivation levels.

**Table 1.** Summary of types of content posted on the student teams’ online project pages.

Type of content posted	Posting Statistic
Team Research Information and Supporting Documentation	Percentage of teams posting content
Research question	95%
Prediction	91%
Experimental design	85%
Conclusion	61%
Team photo	89%
Other images	51%
Research journal files	44%
Data files	39%
Final presentation files	25%
**Comments between teams and mentor**	**Average and range**
Mentor posts to student teams	6.5 (1–43)
Student posts (all team members) to mentor	12.5 (1–124)

**Figure 1.**
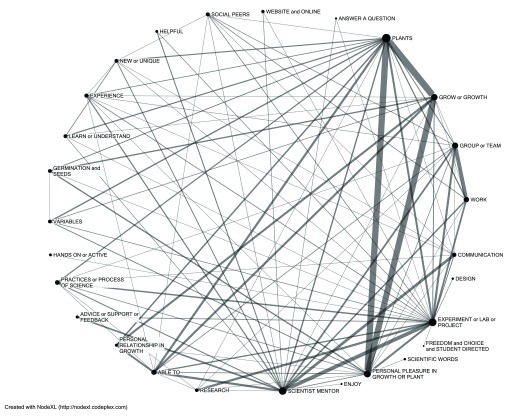
Lexical web illustrating the four themes that students liked most and connections among favored elements. The size of the node represents the number of total respondents liking that element, and the width of the line between nodes is weighted by the number of shared responses. Items with fewer than 20 respondents are filtered out to simplify the lexical web. The size of the nodes corresponds to the number of responses in a given category, while the thickness of the lines represents the number of links between categories.

Across all survey results, students mentioned most frequently four themes as favored elements: plants (26.9%), scientist-mentor (20.3%), growing (15.7%), and experiments (13.8%). While some students responded to the open-ended question by noting only one thing that they liked most, other comments mentioned multiple elements in the same sentence(s). Relationships between themes mentioned by students illustrate a complex network of the four major nodes and how they are interconnected and also cross-linked to other favored elements ( Figure 1). We next narrowed the selection for a view of the networks associated with each of the four major nodes in turn.

**Figure 2.**
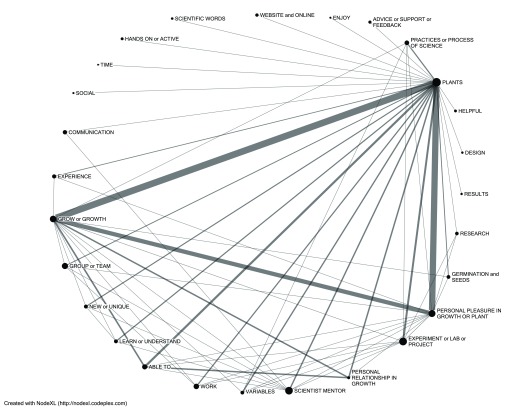
Lexical web illustrating connections of students’ comments about liking the plants to other favored elements. The size of the node represents the number of total respondents liking that element, and the width of the line between nodes is weighted by the number of shared responses. Items with fewer than 10 respondents are filtered out to simplify the lexical web.

What many students explicitly liked about plants was growing them, although the comments about liking plants formed a relatively dense web of connections to other items ( Figure 2). Looking closely at the language that students use about plants, we saw two subtle, distinct descriptions about interactions with their study organisms. Students expressed a sense of enjoyment as a result of their interaction with plants, which was often connected to closely observing their plants: *“I enjoyed seeing my plants grow and display their traits.*” Less commonly but importantly students expressed a personal relationship, often a personal responsibility, for tending to their plants. (1) *“Working with and caring for the plants was my favorite part*.” (2) *“The caring and effort you had to put into it. It was kind of like babysitting a child you could say. Because just like a child you had to watch and care for it.”* Students mentioned liking the plants together with a wide array of other aspects of the PlantingScience experience such as the procedural aspects of manipulating variables and the social aspects of working with friends, classmates, and scientists. Taken together student comments about liking “plants” and “growing” account for the majority of favored program elements.

**Figure 3.**
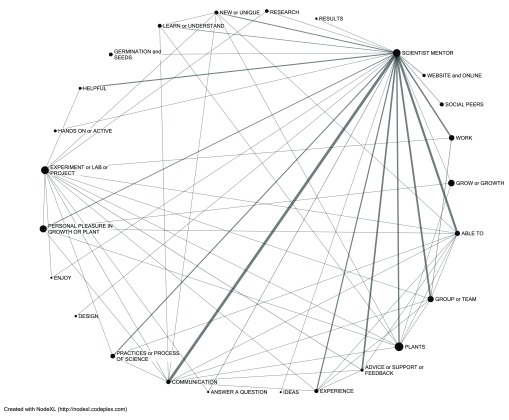
Lexical web illustrating connections of students’ comments about liking their mentors to other favored elements. The size of the node represents the number of total respondents liking that element, and the width of the line between nodes is weighted by the number of shared responses. Items with fewer than 20 respondents are filtered out to simplify the lexical web.

The network of comments that students made about liking most their mentor shows strong connections to many aspects of doing and learning science as part of a science community ( Figure 3). When commenting about liking their mentor, students also noted the uniqueness and the global viewpoint of the online experience. For example, comments included references to experiments, answering a question, communication, advice, team, help, and advantages. Three student quotes that illustrate several of these connections in context are: (1) *“It was great working with a scientist who took the time to give us meaningful feedback. It really helped me learn and experiment.*” (2) *“The ability to interact with real life scientists was interesting and unprecedented in my life. Our group bonded with our mentor and it provided an awesome experience overall, as well as the chance to create and enact our own experimental design.”* (3) *“I liked having the advantage to speak with scientists from other areas around the world and looking at other experiments being done by other students. This to me helped give my group and I more ideas for our experiment and it kind of showed us how we could improve ours and make it a little more detailed with less problems.*”

**Figure 4.**
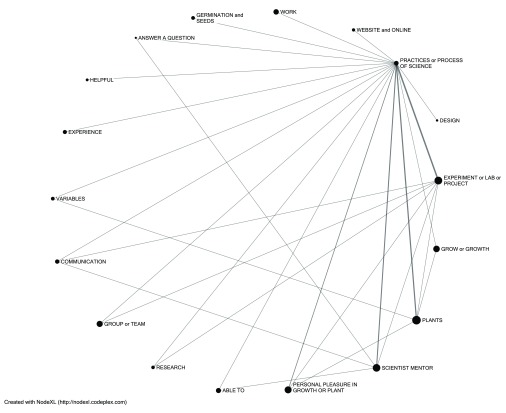
Lexical web illustrating connections of students’ comments about liking the experiment to other favored elements. The size of the node represents the number of total respondents liking that element, and the width of the line between nodes is weighted by the number of shared responses. Items with fewer than 10 respondents are filtered out to simplify the lexical web.

Although students less commonly cited liking most the experience of engaging in scientific practices and experiments, comments on this fourth major theme were also connected to the mentor, the collaborative team research, and the liberation that student-led inquiry offers ( Figure 4). Phrases such as “we got to” or “I was able to” or “had the freedom to” were common signs that students valued the ownership of their research project and ideas. Students appreciated getting to choose variables, particular techniques, particular species of plants as subjects, and the research question. Students also valued the combination of independence and collaboration of the environment in terms of working with and learning from others. Two student quotes capture both of these themes: (1) *“It was interesting to see what kinds of experiments different people came up with, and how they went about testing their hypothesis. I also like coming up with our idea in itself,”* (2) *“I liked how personal it was with our mentor, I also really enjoyed the freedom we had on deciding what we wanted to do and how we wanted to do it. We just picked our project then we tested everything ourselves and planned out everything and presented it just like real scientists would.”*

## Discussion

The aggregated responses provide a picture of the major features that students appreciate most from the blended learning experience of conducting team-led plant investigations in their classrooms and collaborating with domain experts and peers online. Students highly value communicating with their mentors; it both creates a personal connection and provides students with a contextualized experience, working as part of a scientific community. Student comments about scientific practices demonstrated the importance they place on ownership of their own learning and the value of integrating authentic practices like data collection and interpretation into the experience. This study also indicates that students highly value, perhaps most of all, the interactions they have with plants as their study organisms.

As an examination of student attitudes toward program design features, this analysis suggests that key objectives are being met and it hints to some challenges for digital learning environments. Many students responded favorably to the design features that promote experiential and collaborative learning with plants, connections to scientists, and digital opportunities for feedback and reflection. A previous study documented that participating scientists use an array of mentoring techniques including socializing students into science, modeling scientific thinking, and combining content and practices naturally ( Adams & Hemingway, 2014). Here we see that participating students respond by expressing appreciation for the personal relationships they form with scientists, plants, and peers while working as members of a research community. As digital connection to domain experts was a key program intervention, the stronger student response to plants than mentors warrants discussion and further exploration. Students interacted daily with plants and intermittently with their mentors during the course of the team investigations. Challenges of asynchronous discourse with mentors that likely play a role include communication delays, computer access and school schedules and the classroom being a “black box” to some scientists. The frequency of exposure may also influence the high frequency with which students cited liking plants. We would argue the students’ strong positive attitude towards plants as their study subjects reflects an authentic trajectory for developing scientists. Mentors may open the door to the scientific enterprise, and once through it is the discovery process that captures students’ intellectual curiosity. Mentors, advisors, sponsors—more senior experts by any name and at any stage of the career path—are facilitators or cultivators, not generators, of individual wonder and talent.

Prior studies have shown that students studied in several countries find plants less interesting than animals, uninteresting, or downright boring ( Fancovicova & Prokop, 2010; Kinchin, 1999; Randler *et al.*, 2012). While our study was not designed to test students’ relative interest in plants versus animals, our findings of students’ strongly positive attitude about working with plants in this learning setting indicates that how students are exposed to plants matters a great deal. This view is not new ( Uno, 2009), and it is reinforced by other studies. For example, learning experiences that emphasized observations in the local environment enhanced interest in both plants and animals among Swiss precollege students ( Lindemann-Mathhies, 2005). Similarly, improvements in student attitudes towards plants accompany shifts in curricular approach to student-centered, active learning in an undergraduate botany course ( Goldberg & Ingram 2011; Ward *et al.*, 2014). The appreciation and caring relationships that students expressed for plants in this study invoke a sense of biophilia, which Wilson (1984) describes as the human urge to affiliate with other forms of life. To counter ‘plant blindness’ students need opportunities to experience the lives of plants: *“this experience taught me that plants are more than what they just appear to be, they are creatures that develop in ways that are so different than mammals, humans, etc. they are a very beautiful type of species and they are very amazing to learn about.”*

Students’ attitudes, learning, achievement, and career path are linked in close and complex ways ( Osborne *et al.*, 2003). Analyses of attitudes about science from open-ended text analysis are less common than analyses based on Likert scale responses and pose unique challenges compared to measurement of student learning ( Lovelace & Brickman, 2013). While there is a clear need for assessments that produce evidence of student content knowledge and proficiency in science practices ( National Research Council, 2014), the affective domain is a powerful and under-utilized body of evidence in the development of student learning ( Trujillo & Tanner, 2014). In this analysis we began to tease out some of the indicators that students self-report as being positive to their learning experience. We see these as potentially important to the development of future instructional materials and activities and as an indicator of the effectiveness of the PlantingScience program.

These findings have implications for other projects that use technology to support more authentic science practices in science classrooms. Just doing an investigation is not sufficient for deep learning ( Bell *et al.*, 2003) and digital tools for collaboration and communication used effectively can enhance student motivation and understanding ( Mistler-Jackson & Songer, 2000). Digital learning environments generally accumulate large amounts of data rapidly. Without being prohibitively time consuming, the text-analysis approach allowed us to reveal broad patterns in a large set of open-ended data. Students participating in PlantingScience appear to give primacy to the personal science experience with the digital collaboration serving as an enhancer of the experiential learning. Placing students in an environment where they are asked to behave and think like scientists as they conduct investigations, while at the same time providing a mentor who can model scientific thinking, is a powerful combination for students to experience change in their worldview about science, scientists, and plants.

## Data availability

Dataset 1. Student Responses. http://dx.doi.org/10.5256/f1000research.6223.d44181 ( Hemingway *et al.*, 2015).
