# Supplementary material for: Digital collaborative learning: identifying what students value
Source: F1000Res. 2015 Mar 20;4:74. [Version 1] doi: 10.12688/f1000research.6223.1 (PMC4457119; doi:10.12688/f1000research.6223.1)
Supplement: Peer review history

# Peer review: Referee response for version 1

**DOI:** 10.5256/f1000research.6677.r8776

**Published:** 2015-06-01

**Authors:** Graham Scott

**Affiliations:** 1 Bioscience Education Research Group, School of Biological, Biomedical and Environmental Sciences, University of Hull, Hull, UK

This paper makes an important contribution to the literature about engagement with botany in formal schooling. It presents a project that harnesses the enthusiasm of practicing scientists and the power of the internet to enable children (and their teachers) to learn about plants and practical science in an enabling environment.

The paper presents an interesting lexical analysis of the comments of a very large sample of participating children and draws out conclusions that will be of value to those individuals who care about the botanical (and wider scientific literacy) of the next generation.

I found the discussion around figure 2 and the interaction between the affective domain and cognitive domains in the context of growth particularly interesting. It complements the findings of others who have suggested that pedagogies which enhance this interaction are particularly effective and should be a focus of those of us trying to re-connect children and young people with plants and animals in the natural world.

I do have some very minor suggestions for alterations to the current version of the manuscript that the authors might like to consider:

1. Lexical analysis is not particularly common and I think therefore that it will be unfamiliar to the much of the readership of this paper. For this reason I suggest that the authors might provide a little more detail in the description of figure 1. For example, four key nodes are highlighted in the text (plants; scientist mentor; growing and experiments) but from the figure (at the resolution provided) a reader might see/infer five (plants; scientist mentor; experiment or lab project; personal pleasure in growth or plant; grow or growth; and, group or team). Perhaps a little more text to expand the methods section to explain how the four that are used were chosen (what cut off criterion was applied for example) and how these relate to the figures would be helpful.
2. I feel that the sentence “The network of comments that students made about liking most their mentor shows strong connections …” is a little difficult to follow and suggest that it might be helpfully re-worded to improve the clarity of the message.

I have read this submission. I believe that I have an appropriate level of expertise to confirm that it is of an acceptable scientific standard.

# Peer review: Referee response for version 1

**DOI:** 10.5256/f1000research.6677.r8133

**Published:** 2015-04-07

**Authors:** Melanie Link-Perez

**Affiliations:** 1 Department of Biology, Armstrong State University , Savannah, GA, USA

This article by Hemingway and co-authors presents the results of a lexical analysis of an open-ended survey question presented to secondary school students participating in the PlantingScience ( www.plantingscience.org) program. The survey question probed what students most-liked about participation in the inquiry-based classroom research experience supported by online discourse with a scientist mentor.

The article is a well-written and, in my opinion, important contribution to the literature about science education in general and botanical education in particular. The major themes that emerged from the research are that students value their personal relationships both to their scientist mentor and their study organisms, and that the ability to exert some control over choice of study organism, research question, or methodology encouraged students to take ownership in their learning experience. The program’s intervention of connecting student researchers with plant scientist mentors via online discourse seems particularly important given the lack of botanical training of many primary and secondary teachers and the significance of a plant mentor for stimulating lifelong interest and awareness of plants. This is an important article for increasing visibility of the PlantingScience program, but the findings have broader implications for botanical education; in particular, it provides a strong example of the value of directly exposing students to the lives of plants as an effective means of replacing plant blindness with plant appreciation and interest.

Some general comments:

- Abstract is concise and clear; provides adequate context for study and presents the major conclusions.
- Discussion related to Figure 4 was illuminating and suggests ways that educators can encourage students’ interest in inquiry-type projects of any discipline.
- The combination of the lexical webs and example student comments was an effective illustration of the major themes identified by the analysis.
- Excellent selection of student quotes.
- Methods used for analysis of survey data are sound and the conclusions are justified by the data.

The paper generally is clearly written but could be improved by making some of the text more explicit. For example, consider this sentence: “For plant science, meaningful and early exposure may be critical…”  As a reader, I am unclear about what the authors are implicitly stating here, and it would be better for them to be explicit about their meaning. When I read, “For plant science” in that sentence, I am left feeling that something is missing, that this thought is incomplete (What about plant science? For plant science to do what? For plant science to be considered important, valid, of interest? Critical for what?). The writing will be stronger when the connections are explicitly and completely stated.

Please elaborate on “cross-cutting concepts” in the reference to the National Research Council (2009) (“cross-cutting concepts applied in innovative solutions”), since this meaning is unclear.

I refrained from reading with an eye toward grammatical issues, but I would like to make one suggested revision: “The online platform ( www.plantingscience.org) not only eliminates geographic limitations but also makes student thinking visible through its design features, enabling students, teachers and mentors to monitor thinking and learning and to provide feedback.”

Thank you for the opportunity to review this excellent paper.

I have read this submission. I believe that I have an appropriate level of expertise to confirm that it is of an acceptable scientific standard.
